# Evaluation of a Catalyst Durability in Absence and Presence of Toluene Impurity: Case of the Material Co_2_Ni_2_Mg_2_Al_2_ Mixed Oxide Prepared by Hydrotalcite Route in Methane Dry Reforming to Produce Energy

**DOI:** 10.3390/ma12091362

**Published:** 2019-04-26

**Authors:** Carole Tanios, Cédric Gennequin, Madona Labaki, Haingomalala Lucette Tidahy, Antoine Aboukaïs, Edmond Abi-Aad

**Affiliations:** 1 Unité de Chimie Environnementale et Interactions sur le Vivant (UCEIV, E.A. 4492), MREI, Université du Littoral Côte d’Opale (ULCO), F-59140 Dunkerque, France; caroletanios@outlook.com (C.T.); cedric.gennequin@univ-littoral.fr (C.G.); lucette.tidahy@univ-littoral.fr (H.L.T.); antoine.aboukais@univ-littoral.fr (A.A.); edmond.abiaad@univ-littoral.fr (E.A.-A.); 2 Laboratory of Physical Chemistry of Materials (LCPM)/PR2N, Faculty of Sciences, Lebanese University, Fanar, P.O. Box 90656, Jdeidet El Metn, Lebanon; 3 Lebanese Atomic Energy Commission (CLEA), National Council for Scientific Research (CNRS), P.O. Box 11-8281, Riad El Solh 1107 2260, Lebanon

**Keywords:** dry reforming, hydrogen, methane, carbon dioxide, toluene, cobalt, nickel, hydrotalcite

## Abstract

Ni, Co, Mg, and Al mixed-oxide solids, synthesized via the hydrotalcite route, were investigated in previous works toward the dry reforming of methane for hydrogen production. The oxide Co_2_Ni_2_Mg_2_Al_2_ calcined at 800 °C, Co_2_Ni_2_Mg_2_Al_2_800, showed the highest catalytic activity in the studied series, which was ascribable to an interaction between Ni and Co, which is optimal for this Co/Ni ratio. In the present study, Co_2_Ni_2_Mg_2_Al_2_800 was compared to a commercial catalyst widely used in the industry, Ni(50%)/Al_2_O_3_, and showed better activity despite its lower number of active sites, as well as lower amounts of carbon on its surface, i.e. less deactivation. In addition to this, Co_2_Ni_2_Mg_2_Al_2_800 showed stability for 20 h under stream during the dry reforming of methane. This good durability is attributed to a periodic cycle of carbon deposition and removal as well as to the strong interaction between Ni and Co, preventing the deactivation of the catalyst. The evaluation of the catalytic performances in the presence of toluene, which is an impurity that exists in biogas, is also a part of this work. In the presence of toluene, the catalytic activity of Co_2_Ni_2_Mg_2_Al_2_800 decreases, and higher carbon formation on the catalyst surface is detected. Toluene adsorption on catalytic sites, side reactions performed by toluene, and the competition between toluene and methane in the reaction with carbon dioxide are the main reasons for such results.

## 1. Introduction

With the development of technology and the growth of societies, the amount of waste is growing at an exponential rate [1,2]. Thus, the pollution of the planet by man-made waste has become a global problem. The major challenges facing the world today include the need for renewable sources of energy and the reduction of greenhouse gas emissions, which contribute to global warming, and are due to limited energy sources and environmental protection [3,4,5]. Faced with all these problems, biomethanation has the dual benefit of being simultaneously a renewable energy production sector and an alternative waste treatment route. Biomethanation is a natural biological method based on the activity of a microbial flora that converts organic matter into more simple compounds. This process produces digestate, which can be used as fertilizer for soils, and a gas, called biogas, which can be used as a green source of alternative energy to replace fossil fuels [6]. The coupling of biogas production with catalytic processes makes it possible to convert the latter into higher value chemical compounds. For example, hydrogen is considered a clean and renewable source of energy. The hydrogen and carbon monoxide formed by the reforming of biogas make it possible to obtain a wide range of chemical compounds such as formaldehyde, acetic acid, and liquid hydrocarbons [7,8,9]. 

As a result, the reforming reaction between methane and carbon dioxide, which is called the dry reforming of methane, or DRM (CO_2_ + CH_4_ → 2 CO + 2 H_2_, Δ*H*^0^_298K_ = 247 kJ.mol^−1^ (a)), is a highly strategic industrial goal today not only to reduce greenhouse gas emissions and to valorize the biogas, but also to produce the synthesis gas (CO, H_2_) in order to elaborate fuels. DRM is an endothermic reaction but needs suitable catalysts to induce sufficient conversions, even at higher temperatures [10]. 

Most research studies in reforming have been done with a gas mixture model without taking into account the contaminants present in the biogas [11]. However, these contaminants, even in trace amounts, can lead to poisoning of the metallic phase of the catalyst [11]. For example, several works performed in the presence of sulfur compounds showed the deactivation of catalysts even with very low levels of these compounds. Chattanathan et al. [12] studied the effect of adding different concentrations of H_2_S (0.5 mol%, 1 mol%, and 1.5 mol%) during biogas reforming. They found that CH_4_ and CO_2_ conversions decreased by approximately 20% after 10 min of testing, even after the introduction of the lowest amount of H_2_S. Chiodo et al. [13] showed a complete deactivation of the Ni-based catalysts, with different rates, when adding 1 to 2 ppm of H_2_S. Appari et al. [14] proposed a detailed kinetic model that was capable of simulating the reforming of biogas on Ni-based catalysts in the presence of H_2_S. They reported that for temperatures about 700 °C, complete deactivation of the catalyst takes place. Jablonski et al. [15] showed that Ni catalysts were completely deactivated under 1 ppm, 3 ppm, and 5 ppm of different sulfur species. They postulated that the effect of sulfur poisoning is so strong and that the use of hydrocarbon fuels requires the removal of sulfur to reach levels lower than 1 ppm. 

Thus, it will be important to study the effect of the compounds considered as impurities that exist in real biogas, besides CH_4_ and CO_2_ [11,16]. These impurities are classified into four categories: nitrogen compounds, sulfur compounds, terpenes, and other volatile organic compounds (VOCs) [17]. For a better interpretation of the results, it is preferable first to study the effect of a model molecule on catalytic DRM. Knowing that sulfur compounds are already widely studied, we will focus on VOCs, selecting toluene (C_6_H_5_–CH_3_) as a test molecule. Indeed, we performed a chemical analysis of real biogas and confirmed the presence of the VOC toluene among the impurities [18]. 

In a previous work [19], the catalytic performance of Co_x_Ni_y_Mg_z_Al_2_800 oxides (x and y = 0, 1, 2, 3, 4; z = 2, 4; x + y + z = 6; x, y, and z are the nominal molar ratios) synthesized via the hydrotalcite route and calcined at 800 °C was investigated toward the DRM. Co_2_Ni_2_Mg_2_Al_2_800 proved to be the most active due to the stoichiometry of Co and Ni in this oxide, which are suitable for the formation of a Co–Ni alloy known for its reactivity in the studied reaction. 

In the present work, Co_2_Ni_2_Mg_2_Al_2_800 is compared to a commercial catalyst that is widely used in the industry: Ni(50%)/Al_2_O_3_. The stability, during 20 h, of Co_2_Ni_2_Mg_2_Al_2_800 in the dry reforming of methane is also studied. In addition to this, toluene is introduced into the catalytic test to assess its impact as an impurity present in biogas. This work appears to be the first attempt to evaluate toluene poisoning on Co–Ni catalysts in the DRM. Physicochemical characterization was carried out on the catalysts before and after each test, with and without toluene.

## 2. Experimental Section

### 2.1. Catalyst Preparation

Co, Ni, Mg, and Al-based mixed oxide was synthesized by the hydrotalcite route with a molar ratio M^(II+)^/Al^3+^ = 3 [19]. A solution containing appropriate quantities of Co(NO_3_)_2_·6H_2_O, Ni(NO_3_)_2_·6H_2_O, Mg(NO_3_)_2_·6H_2_O, and Al(NO_3_)_3_·9H_2_O was added dropwise, under vigorous stirring, to an aqueous solution of NaOH (2 M) and Na_2_CO_3_ (1 M). The pH was maintained at 9, and the temperature was maintained at 60 °C. The resulting slurry was heated at 60 °C for 18 h to slowly crystallize the hydrotalcite phase. Then, the precipitate was filtered, washed several times with hot deionized water (60 °C) until a neutral pH is obtained, and dried at the same temperature for 48 h. The precipitate was ground to obtain fine powder and then calcined at 800 °C for 4 h under a flow of air (2 L·h^−1^, 1 °C·min^−1^). The calcined solid at 800 °C was named Co_2_Ni_2_Mg_2_Al_2_800, where the subscripts indicate the nominal molar ratio.

A commercial catalyst that is widely used in industry, NiO/Al_2_O_3_, where the Ni weight percentage is 50, was purchased from Acros Organics and used as provided. It will be designated by Ni(50%)/Al_2_O_3_. 

### 2.2. Characterization of the Solids

The structure of the solids was analyzed at room temperature by X-ray diffraction (XRD, Bruker D8) technique in a Bruker D8 advance diffractometer equipped with a copper anode (λ = 1.5406 Å). The scattering intensities were measured over an angular range of 20° < 2θ < 70° with a step-size of 2θ = 0.02° and a count time of 2 s per step. The diffraction patterns have been indexed by comparison with the joint committee on powder diffraction standards (JCPDS) files. This comparison was realized by EVA software. 

The crystallite size was calculated from the broadening of the main diffraction lines using the Scherrer equation [20]:

(1) $$L=\frac{0.9\lambda }{\beta \mathrm{Cos}\theta }$$

where L is the crystallite size, λ is the X-ray wavelength (λ = 1.5406 Å), β is the line broadening, and θ is the Bragg angle. 

The reducibility of the calcined catalyst was measured by the temperature programmed reduction (TPR) method with an Altamira AMI 200 apparatus. A sample quantity of 50 mg was activated in a quartz tube from ambient temperature to 150 °C under argon with a flow rate of 30 mL.min^−1^ and kept at 150 °C for 1 h to remove any physisorbed substances. Then, the sample was cooled to 30 °C in argon before being heated to 950 °C under a flow of 5% H_2_/Ar at a rate flow of 30 mL.min^−1^ with a heating rate of 5 °C·min^−1^. The hydrogen consumed during the reduction was detected by a thermal conductivity detector (TCD).

Temperature programmed oxidation (TPO) is carried out on the catalysts after each test with the same Altamira AMI 200 device, following the evolution of the oxygen consumption as a function of the temperature. The TPO is used to determine the nature and amount of carbon deposited during the catalytic reaction. First, 20 mg of calcined sample at 800 °C is treated at 150 °C for 1 h under a helium flow of 30 mL.min^−1^, after a rise from room temperature at a rate of 10 °C·min^−1^. Then, the TPO analysis were carried out under a gas flow of 10% O_2_ in helium with a temperature rise of 5 °C·min^−1^from room temperature to 900 °C. The amount of consumed O_2_ is quantified by a TCD.

Differential scanning calorimetry/thermogravimetry (DSC-TG, TA instrument SDT Q600) analyses were done simultaneously on a TA instrument SDT Q600 apparatus. Two alumina crucibles are symmetrically put on a support inside a furnace. The first crucible is empty, representing the reference crucible. Solids are introduced in the other crucible, and then heated from room temperature up to 900 °C at a rate of 5 °C·min^−1^ under an air flow of 75 mL·min^−1^. A thermocouple system controls and measures the temperature of the sample. The measured difference between the sample and the reference allows the thermal differential (temperature difference) and the gravimetric (loss or gain of weight of the sample) analyses.

### 2.3. Experimental Conditions of the Test

Dynamic catalytic tests were carried out, from 400 °C to 800 °C, in a U-shaped fixed bed quartz reactor equipped with a quartz frit. The reactor was fed with a gas mixture of CH_4_/CO_2_/Ar with respectively 20%/20%/60% at atmospheric pressure so that the molar ratio CH_4_/CO_2_ was one. The total gas flow was 100 mL·min^−1^, the catalyst weight 100 mg, and the gas hourly space velocity ~32 000 h^−1^. The effluent gas was analyzed using a micro gas chromatography Varian CP-4900 equipped with a Poraplot Q (PPQ) column, a molecular sieve, and a TCD.

A system based on the saturation principle was used to introduce toluene. A container filled with pure liquid toluene was kept at constant temperature and pressure. This container is equipped with a perforated plate allowing the continuous controlled diffusion of a small amount of toluene. The diffused product is mixed with the carrier gas, which is argon. The saturator placed in an oven, which allows the control of the quantity of diffused toluene. Thus, a larger amount of toluene will be released as the temperature increases. In order to determine the amount of the liquid product diffused per unit of time as a function of the saturator temperature, a preliminary calibration for toluene was carried out. Concentrations of 1470 ppm and 180 ppm toluene corresponding to saturator temperatures of 70 °C and 30 °C respectively are used in this work. The total flow of the reagents (CH_4_, CO_2_, and toluene) and the carrier gas (Ar) is 100 mL·min^−1^. Then, toluene is vaporized, carried by argon, and mixed with CH_4_ and CO_2_. Heating filament wounds on the stainless steel pipes were used to avoid toluene condensation throughout all the setup. The mixture was sent toward the quartz reactor containing the catalyst and placed in a furnace. The test was performed under atmospheric pressure. For the tests with toluene, the catalysts were pressed under a pressure of 2 tons, and then ground in a mortar. The powders obtained are passed through two superposed sieves with dimensions of 350 μm and 800 μm to obtain particles with uniform size. 

Varian 3800 CPG gas chromatography is used for the analysis of the gas at the reactor outlet. The reactants (CH_4_ and CO_2_) and the products (H_2_ and CO) are analyzed with a thermal conductivity detector (TCD) after separation through a molecular sieve (H_2_ and CO) and a HayeSep Q column (CH_4_ and CO_2_). On the other hand, by-products from the conversion of toluene (C_6_H_6_ and C_2_H_4_) as well as unconverted toluene are analyzed by gas chromatography equipped with a flame ionization detector (FID) and a sep:cpml column PONA CB (this column accurately analyzes paraffins, olefins, naphthalenes, and aromatics in complex hydrocarbon mixtures). The unit is coupled to a mass spectrometer (Omnistar, Pfeiffer vacuum GSD 301 O) to monitor the evolution of products and by-products.

Prior to the reactions (with or without toluene), all catalysts were subjected to a pre-treatment step where they were reduced in situ by a 5 % H_2_ flow at 800 °C for 2 h to activate the catalyst. Then, the reactor was cooled to 400 °C under a flow of argon. 

The conversion rates of CH_4_ (X_CH4_) and CO_2_ (X_CO2_), the selectivity of H_2_ (S_H2_) and that of CO (S_CO_) to obtain their ratio, the yield of each H_2_ (H_2_) and CO (CO), and the carbon balance (CB) were calculated as follows [21,22,23,24]:

(2) $$\mathrm{Methane}\;\mathrm{conversion}\;(X_{{\mathrm{CH}}_{4}}):\;X_{{\mathrm{CH}}_{4}}=\frac{\left({\mathrm{CH}}_{4},\mathrm{in}-{\mathrm{CH}}_{4},\mathrm{out}\right)}{{\mathrm{CH}}_{4},\mathrm{in}}\times 100$$

(3) $$\mathrm{Carbon}\;\mathrm{dioxide}\;\mathrm{conversion}\;(X_{{\mathrm{CO}}_{2}}):X_{{\mathrm{CO}}_{2}}=\frac{\left({\mathrm{CO}}_{2},\mathrm{in}-{\mathrm{CO}}_{2},\mathrm{out}\right)}{{\mathrm{CO}}_{2},\mathrm{in}}\times 100$$

(4) $$H_{2}/\mathrm{CO}\;\mathrm{ratio}:{\mathrm{Ratio}\;H}_{2}/\mathrm{CO}=\frac{S_{H_{2}}}{S_{\mathrm{CO}}}\times 100$$

(5) $$H_{2}\;\mathrm{yield}\;(\%):H_{2}=\frac{H_{2},\mathrm{out}}{2\times \left({\mathrm{CH}}_{4},\mathrm{in}\right)}\times 100$$

(6) $$\mathrm{CO}\;\mathrm{yield}\;(\%):\mathrm{CO}=\frac{\mathrm{CO},\mathrm{out}}{\left({\mathrm{CH}}_{4},\mathrm{in}+{\mathrm{CO}}_{2},\mathrm{in}\right)}\times 100$$

(7) $$\mathrm{Carbon}\;\mathrm{balance}\;(\%):\mathrm{CB}=\frac{\left({\mathrm{CO}}_{2},\mathrm{out}+{\mathrm{CH}}_{4},\mathrm{out}+\mathrm{CO},\mathrm{out}\right)}{\left({\mathrm{CH}}_{4},\mathrm{in}+{\mathrm{CO}}_{2},\mathrm{in}\right)}\times 100$$

In the above formulas, the symbol of the gas designates its volume percentage (proportional to its molar amount), in the inlet (in) or outlet (out) of the reactor.

The experimental error on the values of the conversions is about ±3%.

## 3. Results

### 3.1. Comparison between Co_2_Ni_2_Mg_2_Al_2_800 and the Commercial Catalyst Ni (50%)/Al_2_O_3_

As stated in the introduction, the catalyst that showed the best catalytic activity in DRM in our previous study [19], Co_2_Ni_2_Mg_2_Al_2_800, was evaluated in the present work. Its activity was compared to that of a commercial catalyst widely used in the industry, Ni(50%)/Al_2_O_3_ (Acros Organics).

**Figure 1:**
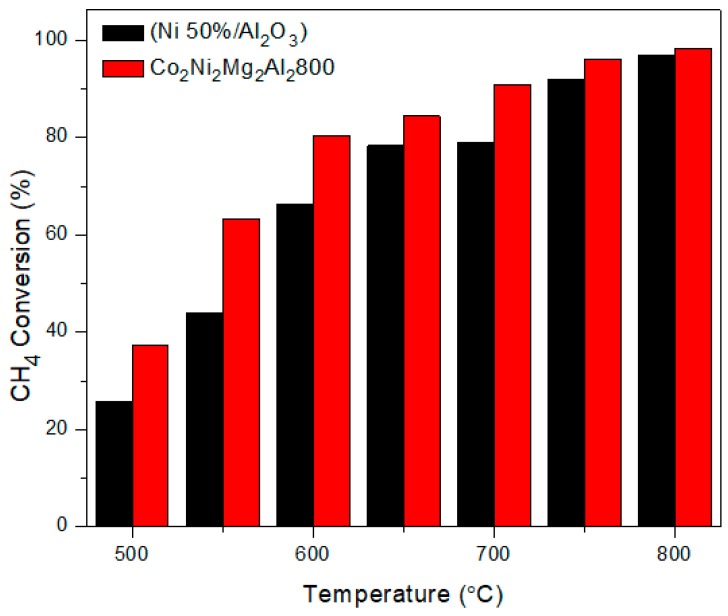
Evolution of the methane conversion in the dry reforming of methane (DRM) versus the reaction temperature for the catalyst Co_2_Ni_2_Mg_2_Al_2_800 and the commercial catalyst Ni (50%)/Al_2_O_3._

Figure 1 compares the methane conversion versus reaction temperature in DRM for Co_2_Ni_2_Mg_2_Al_2_800 and Ni (50%)/Al_2_O_3_.

Ni (50%)/Al_2_O_3_ shows a lower conversion than Co_2_Ni_2_Mg_2_Al_2_800. This difference is clearly observed between 500–700 °C, even though the mixed oxide prepared in the laboratory contains only 24.5% nickel by weight, while the commercial catalyst contains 50% nickel by weight. In fact, a high metal content leads to the formation of agglomerates that decrease the catalytic activity because some of the active sites do not participate in the DRM reaction [25,26]. In addition, the specific surface area obtained by the (Brunauer, Emmett, and Teller) BET method for the commercial catalyst (145 m^2^·g^−1^) is lower than that of the catalyst prepared in the laboratory (161 m^2^·g^−1^). This observation could also explain the difference in methane conversion percentages between these two catalysts. Furthermore, as noted by several authors [10,27,28,29,30], the effect of the interaction between Ni and Co in Co_2_Ni_2_Mg_2_Al_2_800 favors the dry reforming of methane reaction. These authors obtained good activity for Co–Ni catalysts in the dry reforming of methane and attributed it to the formation of Ni–Co alloys that are characterized by a small particle size of Ni and Co, causing a greater dispersion of particles and strong metal-support interaction (SMSI). 

**Figure 2:**
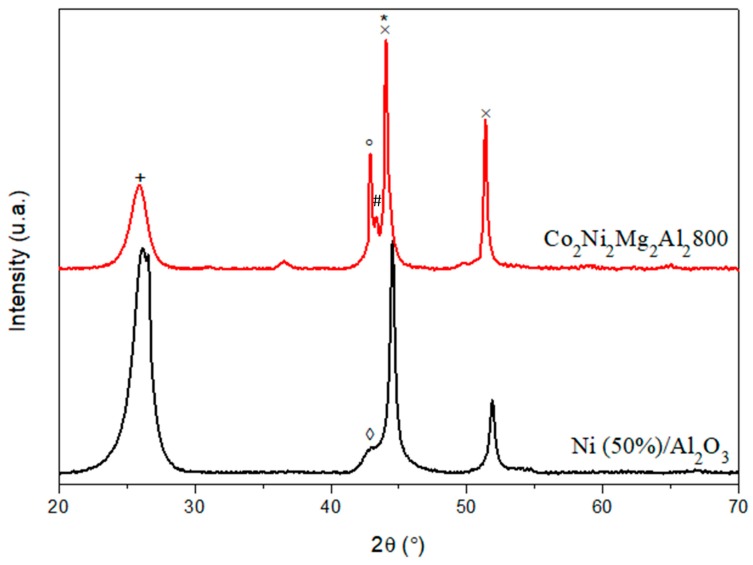
X-ray diffraction (XRD) patterns of Co_2_Ni_2_Mg_2_Al_2_800 and the commercial catalyst Ni (50%)/Al_2_O_3_ after DRM test (* C cubic JCPDS N° 800017; + C hexagonal JCPDS N° 751621; # Co*_2_*AlO*_4_* JCPDS N°380814/CoAl*_2_*O*_4_* JCPDS N°440160/Co*_3_*O*_4_* JCPDS N°421467; × Co JCPDS N°150806/Ni JCPDS N°211152; ° MgO JCPDS N° 450946; ◊ NiAl_2_O_4_ JCPDS N°100339/Al_2_O_3_ JCPDS N°10173).

X-ray diffraction was carried out on these two catalysts after DRM, and the results obtained are given in Figure 2. 

The commercial catalyst Ni (50%)/Al_2_O_3_ shows a diffraction line around 2θ = 43° which corresponds to NiAl_2_O_4_ and does not appear for Co_2_Ni_2_Mg_2_Al_2_800 [31]. In addition, the line around 2θ = 52°, corresponding to the nickel metal resulting from the reduction of Ni oxides (NiO, NiAl_2_O_4_) during the catalytic test, is less intense for Ni (50%)/Al_2_O_3_ than for Co_2_Ni_2_Mg_2_Al_2_800. 

In addition to this, the line at 2θ = 26.8° that corresponds to the hexagonal carbon is more intense for Ni (50%)/Al_2_O_3_, revealing that the amount of crystallized carbon deposited on the surface of this catalyst is greater in this case [32]. 

### 3.2. Stability Test for Co_2_Ni_2_Mg_2_Al_2_800

**Figure 3:**
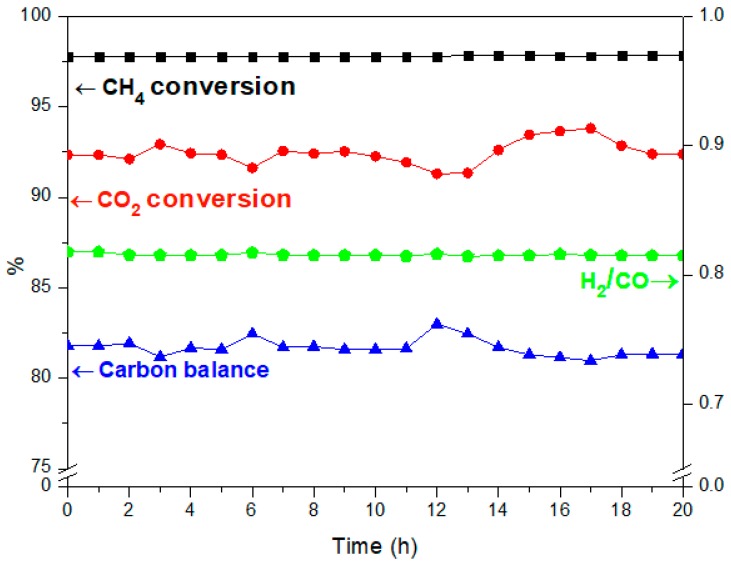
Stability test of Co_2_Ni_2_Al_2_Mg_2_800 in DRM at 800 °C for 20 h.

A good catalyst has to be not only active and selective toward the desired products but also stable over time. In fact, lifetime is one of the most important criteria for choosing a catalyst. In the following, stability tests were carried out on the oxide Co_2_Ni_2_Mg_2_Al_2_800 for 20 h at 800 °C. Figure 3 shows the resulting CH_4_ and CO_2_ conversions, as well as the H_2_/CO ratio and the carbon balance throughout the test.

The conversion of CH_4_ (97%) and the ratio H_2_/CO (0.8) remain constant during the 20-h period, whereas the conversion of CO_2_ and the carbon balance vary respectively between 91–93.5% and 81–83.5%.

These observations indicate that side reactions occur simultaneously with DRM. In fact, the reverse water gas shift (CO_2_ + H_2_ 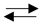 CO + H_2_O) (b) and the methane decomposition (CH_4_ → C + 2 H_2_) (c) compete with the main DRM reaction. Indeed, during the reverse water gas shift (b), CO_2_ reacts with H_2_ to produce CO and H_2_O, thus leading to a ratio H_2_/CO < 1. In fact, small amounts of water were detected. In addition, the methane decomposition (c) produces C and H_2_. Therefore, higher CH_4_ conversion compared to CO_2_ as well as carbon deposition, which leads to a carbon balance lower than 100%, could be due to the occurrence of reaction (c). 

However, despite the occurrence of these side reactions and the carbon deposition produced [19,33,34,35,36,37,38,39], the catalyst Co_2_Ni_2_Mg_2_Al_2_800 was found to be resistant to deactivation during these 20 h, maintaining conversions of CH_4_ and CO_2_ greater than 90%.

Several authors have performed such stability tests on similar catalysts. Serrano-Lotina et al. [40,41] consistently achieved higher CO_2_ conversions than CH_4_ conversions with an H_2_/CO ratio less than one, and attributed these results to a predominance of the reverse water gas shift (b). Dahdah et al. [22] obtained similar CH_4_ and CO_2_ conversions with a H_2_/CO ratio close to unity during the entire stability test period. The catalysts prepared by Yu et al. [42] showed a decrease in activity during the stability test. This observation was attributed to fast carbon deposition and the contribution of reaction (b), which was responsible for the H_2_/CO ratio being less than one.

**Figure 4:**
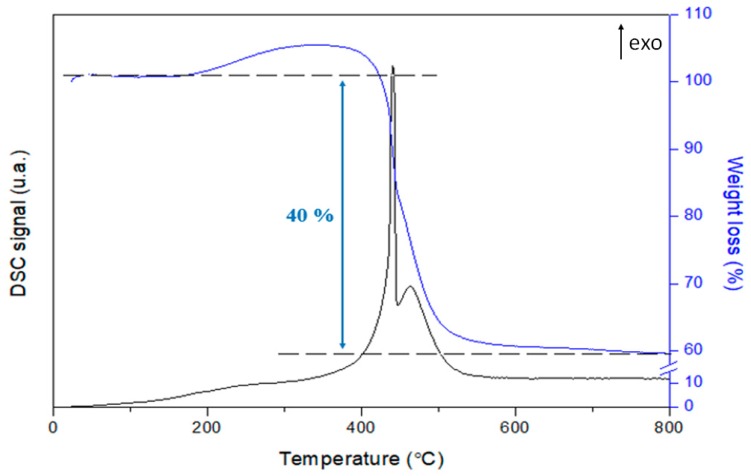
Thermal analysis under air of Co_2_Ni_2_Mg_2_Al_2_800 after stability test for 20 h at 800 °C.

Figure 4 shows the DSC signal and the weight loss recorded for the catalyst Co_2_Ni_2_Mg_2_Al_2_800 after 20 h under stream at 800 °C.

The weight gain observed between 200–400 °C is attributed to the oxidation of the reduced Co and Ni metal particles [42]. The amount of carbon deposited is considerable, and presents 40% of the weight loss. These carbon deposits are oxidized between 440–460 °C (two exothermic DSC peaks) in the presence of air [33], and are probably similar to C_β_-type carbon deposits located close to the metal sites of the catalyst [32,34]. This type of carbon could cover metal particles rendering them inaccessible to the reactants. Furthermore, in many studies on catalyst stability [22,33,43], the authors attribute good activity to a periodic cycle of carbon deposition and removal. This cycle is observed in our case, with a small percentage range varying between 91–93.5% and 81–83.5% respectively for the conversion of CO_2_ and the carbon balance. Other authors also attribute the good stability of the bimetallic catalyst Ni–Co to (i) the interaction between Ni and Co, (ii) their strong interaction with the other chemical elements of the catalyst, (iii) the small size of their particles, and (iv) their high dispersion; thus preventing sintering [23,28,41,44]. Then, it is inferred that the stability of Co_2_Ni_2_Mg_2_Al_2_800 during 20 h under stream, despite the formation of a noticeable amount of carbon due to side reactions, is due to the occurrence of the carbon deposit/removal cycle and to the benefit of the simultaneous presence of Ni and Co in a suitable ratio. 

### 3.3. Dry Reforming of Methane in Presence of Impurities

#### 3.3.1. Influence of Catalyst Shaping

Our previous studies have been carried out on catalysts in powder form [19]. With the introduction of toluene, the catalysts are prepared in the form of pellets to avoid clogging the reactor. The catalysts are pressed and sieved as mentioned in the experimental part. In order to evaluate the influence of the catalyst form (powder or pellet) on catalytic performance, a test without toluene is carried out on pellets and compared to the test on powder. 

**Figure 5:**
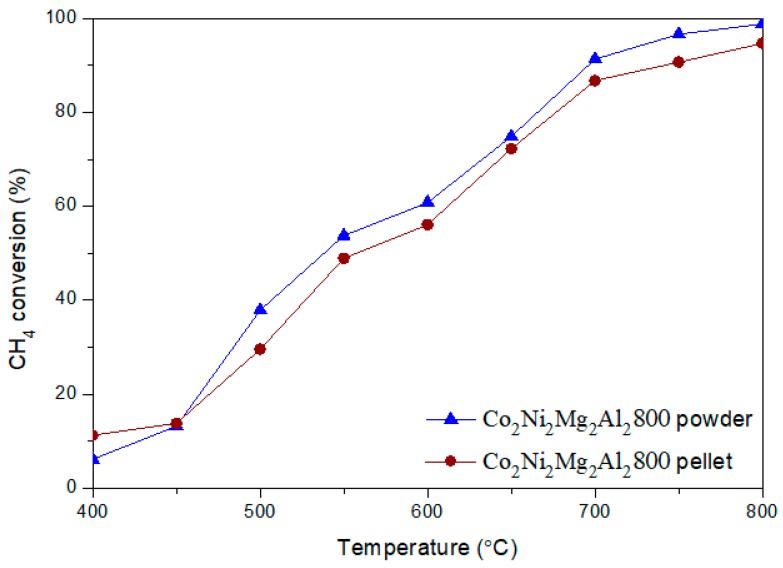
Evolution of the conversion of methane in DRM versus reaction temperature for the two different forms (powder and pellet) of Co_2_Ni_2_Mg_2_Al_2_800.

Figure 5 compares the methane conversion as a function of the reaction temperature for the two different forms (powder and pellet) of the Co_2_Ni_2_Mg_2_Al_2_800 catalyst. The conversions of methane obtained in the presence of the catalyst in the form of powder and pellet are similar, with slightly higher conversions for the powder form. No significant changes in catalyst performance were observed during the catalytic test. This indicates that catalyst shaping does not greatly affect the activity under the selected test conditions. Such results are also obtained in previous studies [26,31]. 

#### 3.3.2. Influence of Adding Toluene

In order to study the effect of toluene addition on the catalytic activity in DRM, two toluene concentrations are selected. The first target level, 1500 ppm, corresponds to the sum of all the impurities present in the biogases collected and analyzed in a previous study [18]. The second target level, 200 ppm of toluene, is close to the highest contents found for each impurity [18]. However, in practice, we obtained concentrations of 1470 ppm and 180 ppm toluene that correspond to saturator temperatures of 70 °C and 30 °C, respectively. Co_2_Ni_2_Mg_2_Al_2_800 is tested in DRM with these two toluene concentrations, designated by T1470 and T180.

**Figure 6:**
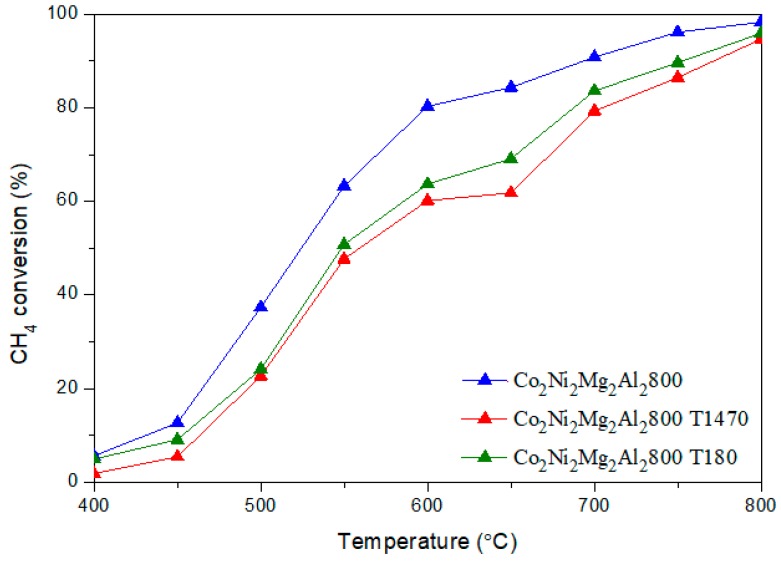
Evolution of the CH_4_ conversion in DRM versus reaction temperature on the catalyst Co_2_Ni_2_Mg_2_Al_2_800 with and without toluene.

**Figure 7:**
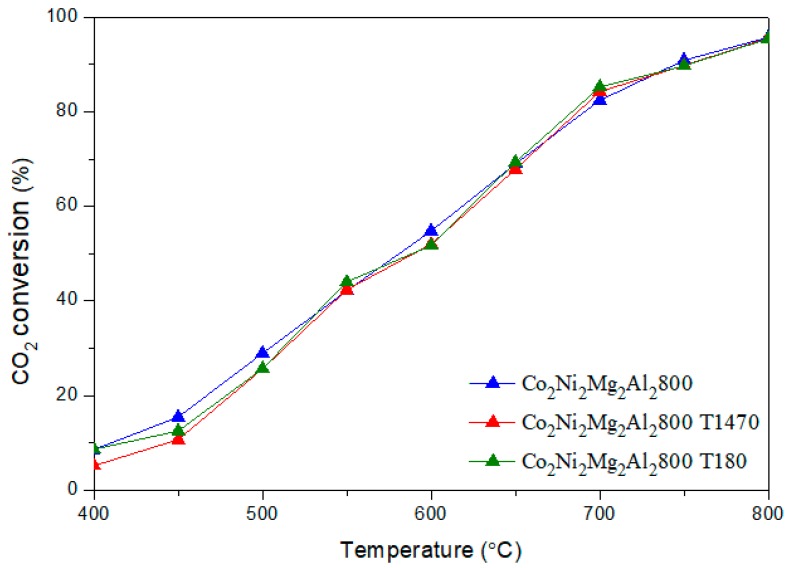
Evolution of the CO_2_ conversion in DRM versus reaction temperature on the catalyst Co_2_Ni_2_Mg_2_Al_2_800 with and without toluene.

Figure 6 and Figure 7 illustrate the CH_4_ and CO_2_ conversions obtained for the two catalysts with and without toluene.

**Figure 8:**
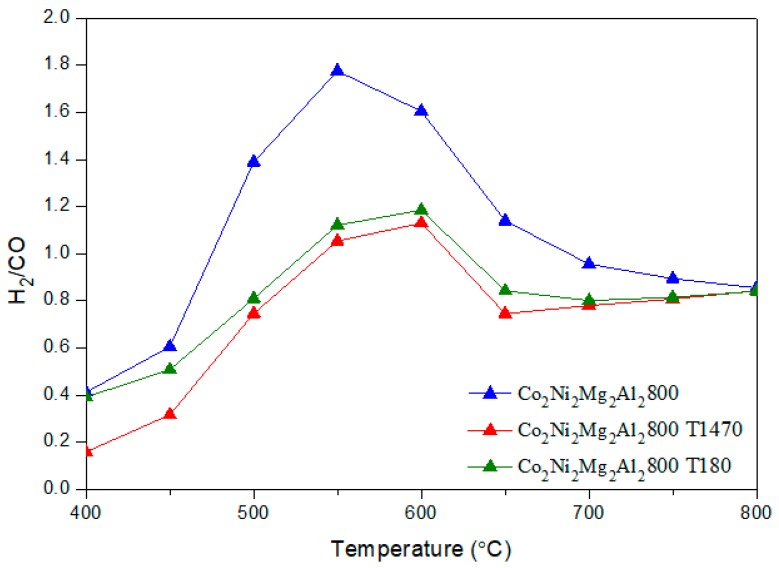
H_2_/CO molar ratio in DRM versus reaction temperature for the catalyst Co_2_Ni_2_Mg_2_Al_2_800 with and without toluene.

**Figure 9:**
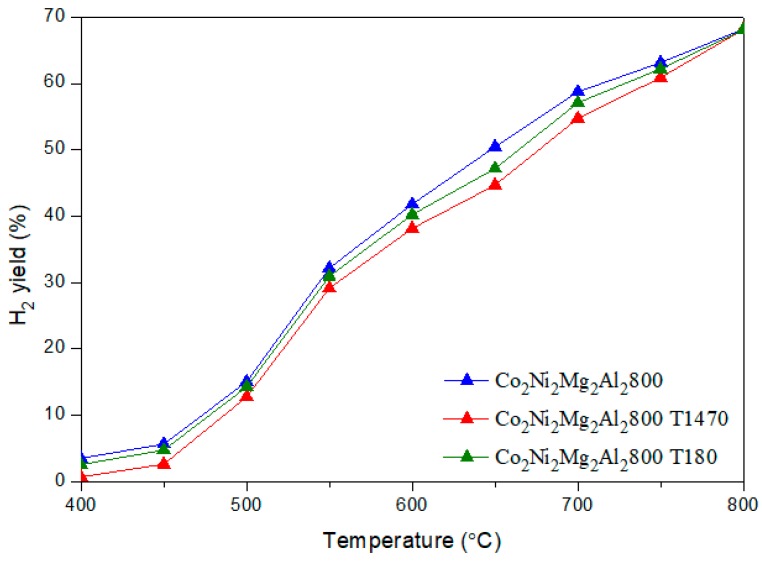
Evolution of the H_2_ yield in DRM versus the reaction temperature for the catalyst Co_2_Ni_2_Mg_2_Al_2_800 with and without toluene.

**Figure 10:**
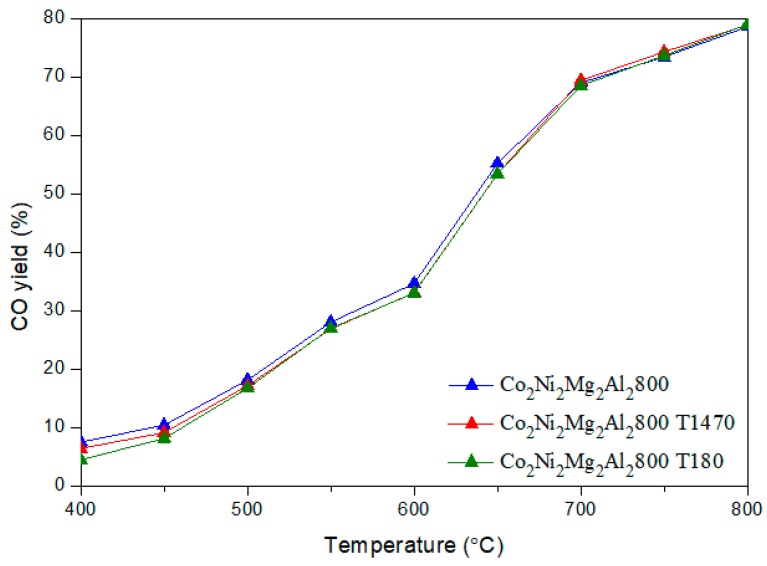
Evolution of the CO yield in DRM versus the reaction temperature for the catalyst Co_2_Ni_2_Mg_2_Al_2_800 with and without toluene.

It is clear that the addition of toluene decreases the conversion of CH_4_ by an average value of 15 %, whatever the reaction temperature. However, the conversion of CO_2_ remains almost the same. The H_2_/CO ratios (Figure 8) obtained in the presence of toluene decrease sharply between 500–700 °C. In addition, the yields of H_2_ and CO (Figure 9 and Figure 10) in the presence of toluene are similar or slightly lower than those in its absence. These results confirm the occurrence of side reactions, in parallel to DRM. These side reactions ((b) to (f)) take place in the absence and presence of toluene, have a strong influence on the catalytic performance toward DRM, and contribute to the reactants or products’ consumption without being strictly accounted for in the equilibrium calculation done for DRM. 

These reactions are as follows [19,33,34,35,36,37,38,39]:

- Reverse water gas shift reaction (RWGS): CO_2_ + H_2_ 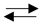 CO + H_2_O  (b) favorable at T > 600 °C
- Methane decomposition: CH_4_ → C + 2 H_2_  (c) favorable at T > 500 °C
- Boudouard reaction: 2 CO → C + CO_2_  (d) favorable at T < 750 °C
- Methanation: CO_2_ + 4 H_2_ → CH_4_ + 2 H_2_O  (e) favorable at T < 750 °C
- Reverse of carbon gasification: CO + H_2_ → C + H_2_O  (f) favorable at T < 750 °C

These reactions lead to more or less consumptions of CH_4_, CO_2_, CO, and H_2_ [19,33,34,35,36,37,38,39]. 

Few authors have studied the effect of hydrocarbons on catalytic DRM. A study conducted by Chiodo et al. [13] reported the influence of several contaminants added to clean biogas on a Ni-based catalyst. Among these contaminants, a mixture of hydrocarbons formed of ethane, ethylene, acetylene, and propylene was studied. With poisoning concentrations below 200 ppm, the catalytic performance obtained was stable for 100 h. Conversely, when a total hydrocarbon concentration of 800 ppm was added to the inlet gas stream, methane conversion was reduced by about 12% after 25 h of testing. The authors attributed the catalytic deactivation phenomenon to carbon formation on the catalyst surface due to hydrocarbons cracking according to the reaction: CnH_2_n → n C + n H_2_. In general, a higher tendency for hydrocarbon cracking occurs as the unsaturation and molecular weight increase [13,45], and therefore aromatics such as toluene are major precursors of carbon. The decomposition of toluene is as follows: C_7_H_8_ → 7 C + 4 H_2_ (g). This reaction is favorable throughout the temperature range used in our catalytic tests (400 to 800 °C). Even for low concentrations of toluene, this reaction occurs in addition to side reactions (b), (c), (d), (e), and (f) largely affecting the catalytic performance in DRM.

In addition, Dagle et al. [46] showed that the presence of benzene and naphthalene on catalysts containing Ni, Rh, and Ir leads to a noticeable decrease in activity due to coking. Similarly, Laprune et al. [45] studied the effect of naphthalene on the conversion of methane, at 700 °C, 800 °C, and 900 °C, on two commercial catalysts based on Rh and Ni. A concentration of 1400 ppm naphthalene poisoned the catalysts strongly during methane reforming. Similarly, the presence of pyrene leads to analogous results, at a concentration of 5 ppm. On the other hand, less resistance is noticed for Ni-based samples in the presence of toluene, and the yield’s decrease is mainly ascribed to the blocking of active sites by carbon.

**Figure 11:**
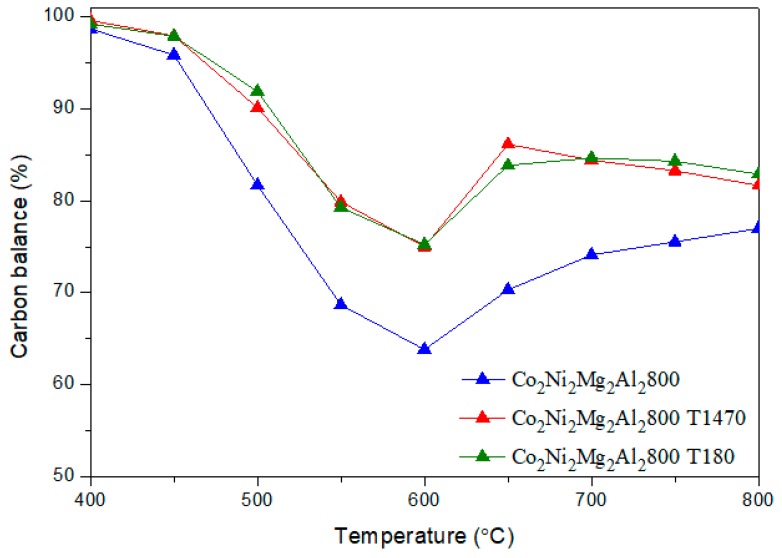
Carbon balance in DRM versus reaction temperature on the catalyst Co_2_Ni_2_Mg_2_Al2800 with and without toluene.

For Co_2_Ni_2_Mg_2_Al_2_800, the evolution of the carbon balance with temperature (Figure 11) shows the same profile in the presence and absence of toluene. However, the balance is less complete in the absence of toluene. This suggests that toluene is not only involved in carbon formation; it also competes with CH_4_ by reacting with CO_2_ according to the reaction of the dry reforming of toluene: C_7_H_8_ + 7 CO_2_ → 14 CO + 4 H_2_ (h) [47]. Reaction (h) could also explain the lower conversions of methane and the lower H_2_/CO ratios obtained in the presence of toluene.

To better understand the mechanism of the reaction taking place in presence of toluene, the evolution of the by-products formed as a function of reaction temperature was followed by a mass spectrometer (MS). The analyses showed the presence of benzene (C_6_H_6_) as a by-product.

**Figure 12:**
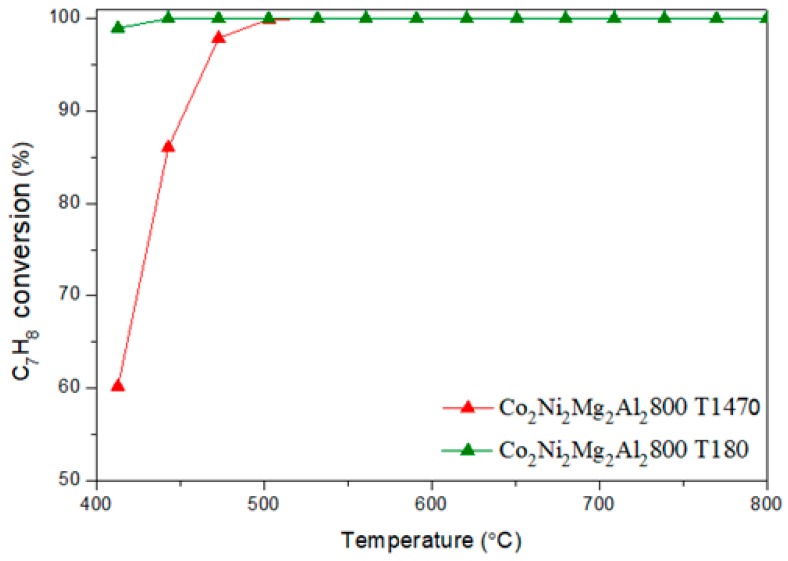
Conversion of toluene in DRM, followed by MS, versus reaction temperature on the catalyst Co_2_Ni_2_Mg_2_Al_2_800 for the toluene contents 1470 ppm and 180 ppm.

**Figure 13:**
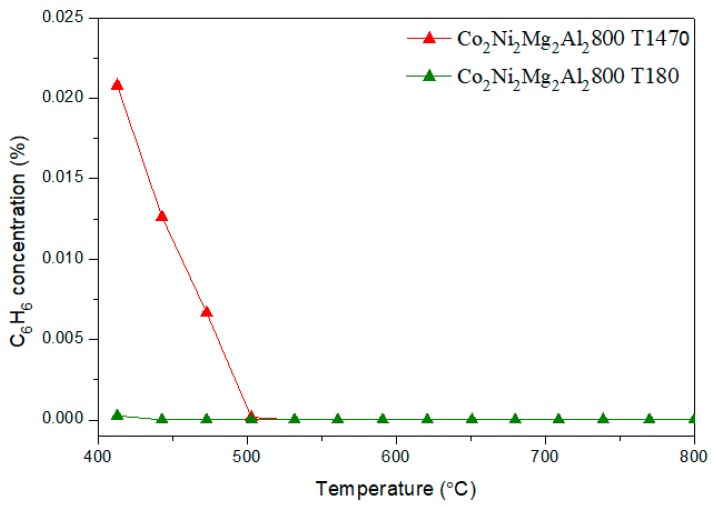
Evolution of benzene, in DRM, followed by MS, versus reaction temperature, on the catalyst Co_2_Ni_2_Mg_2_Al_2_800, for the toluene contents of 1470 ppm and 180 ppm.

Figure 12 and Figure 13 show respectively the conversion of toluene and the evolution of benzene, followed by MS, as a function of the reaction temperature, on Co_2_Ni_2_Mg_2_Al_2_800 for the contents 1470 ppm and 180 ppm of toluene.

We note that the conversion of toluene is complete as soon as the temperature reaches 500 °C. In parallel, the concentration of benzene vanishes completely at 500 °C. Between 400–500 °C, the hydrodealkylation reaction is indeed favorable: C_7_H_8_ + H_2_ → C_6_H_6_ + CH_4_ (i). It consists of the decomposition of toluene into benzene and methane in the presence of the hydrogen formed, which explains the lower conversion of CH_4_ obtained in this temperature range in the presence of toluene compared to in its absence [48].

**Figure 14:**
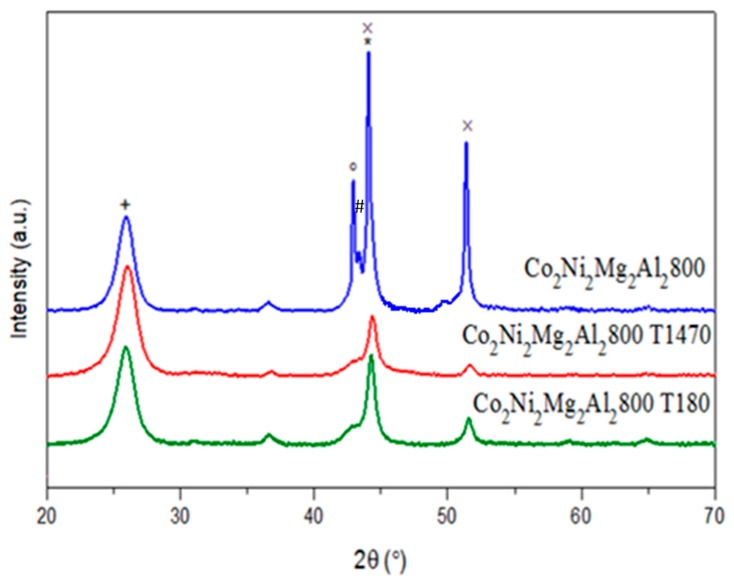
XRD patterns of Co_2_Ni_2_Mg_2_Al_2_800 after test in the presence of toluene (* C cubic JCPDS N° 800017; + C hexagonal JCPDS N° 751621; # Co_2_AlO_4_ JCPDS N°380814/CoAl_2_O_4_ JCPDS N°440160/Co_3_O_4_ JCPDS N°421467; × Co JCPDS N°150806/Ni JCPDS N°211152; ° MgO JCPDS N° 450946).

Figure 14 shows the XRD profiles of Co_2_Ni_2_Mg_2_Al_2_800 after the DRM test in the presence of toluene. 

The intensity of the peaks attributed to carbon (2θ ≈ 26°) is almost the same for Co_2_Ni_2_Mg_2_Al_2_800 and Co_2_Ni_2_Mg_2_Al_2_800 T180 and increases for Co_2_Ni_2_Mg_2_Al_2_800 T1470. However, the intensity of the X-ray diffraction lines corresponding to the Co and Ni metallic phases (2θ ≈ 44 and 52°) and to magnesium oxide (2θ ≈ 43°) decreases in the presence of toluene. In addition, the absence of XRD lines due to AlNi_3_C_0.5_ and AlCo_3_C_0.5_ in the XRD pattern of Co_2_Ni_2_Mg_2_Al_2_800 T180 was noted.

**Figure 15:**
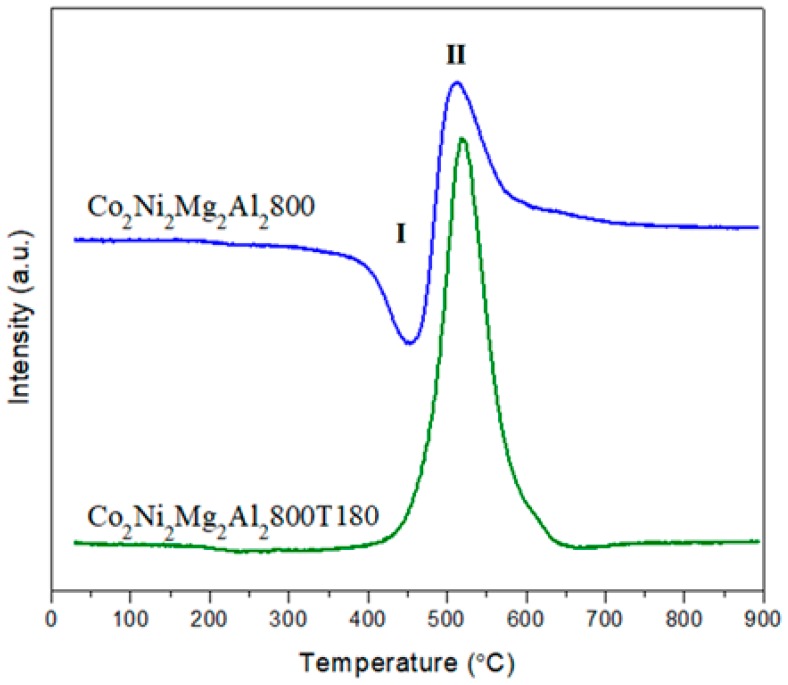
Temperature programmed oxidation (TPO) profiles of Co_2_Ni_2_Mg_2_Al_2_800 after the dry reforming of methane in the absence and in the presence of 180 ppm of toluene.

Furthermore, the TPO profiles (Figure 15) show two peaks, I and II, for Co_2_Ni_2_Mg_2_Al_2_800 and only one peak (peak II) for Co_2_Ni_2_Mg_2_Al_2_800 T180. Peak I is due to the oxidation of metallic Co and Ni according to reactions (j) and (k) [47,49]:

Co(0) + ½ O_2_ → CoO (j)

Ni(0) + ½ O_2_ → NiO (k)

No oxidation was detected for metallic Ni and Co in TPO for Co_2_Ni_2_Mg_2_Al_2_800 T180. In fact, XRD showed a lower amount of metallic Ni for Co_2_Ni_2_Mg_2_Al_2_800 T180 than for Co_2_Ni_2_Mg_2_Al_2_800. 

Peak II in TPO is attributed to carbon oxidation. This oxidation releases carbon dioxide according to the following reaction: C_adsorbed_ + O_2_ → CO_2_; the oxidation peak is more intense for Co_2_Ni_2_Mg_2_Al_2_800 T180 than for Co_2_Ni_2_Mg_2_Al_2_800, suggesting that a higher amount of carbon is deposited on Co_2_Ni_2_Mg_2_Al_2_800 T180. Since the intensity of the XRD line due to carbon is similar for both catalysts, it is suggested that some of the carbon on Co_2_Ni_2_Mg_2_Al_2_800 T180 is present in the amorphous phase and thus not detected by XRD. 

**Figure 16:**
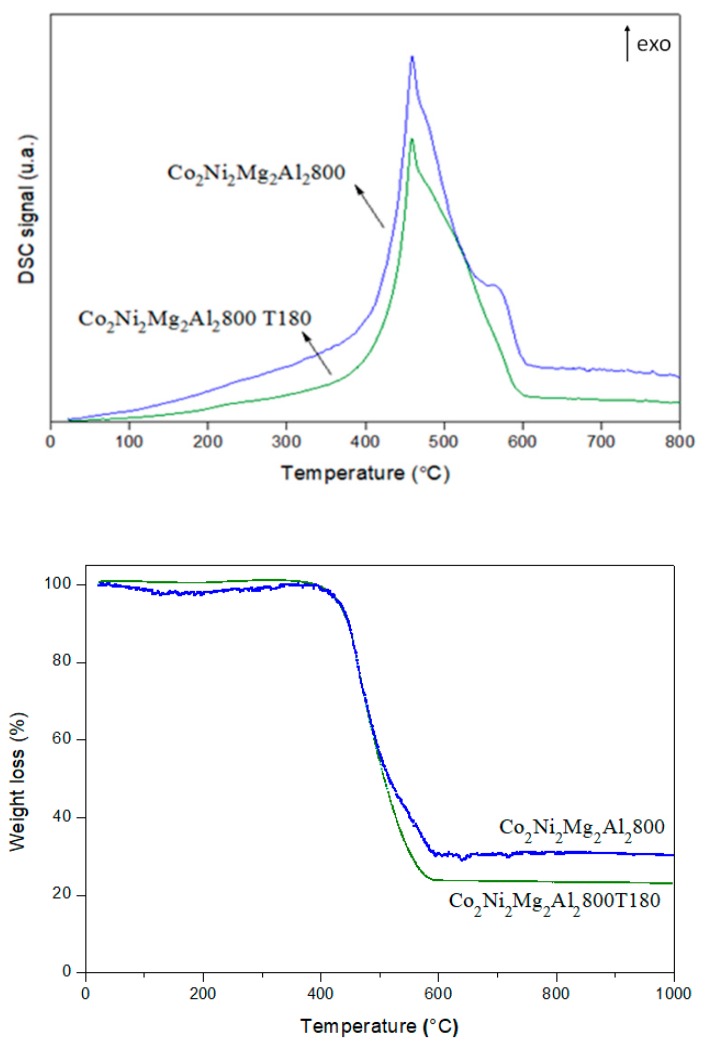
Thermal analysis of Co_2_Ni_2_Mg_2_Al_2_800 after the dry reforming of methane in the absence and the presence of 180 ppm of toluene.

Figure 16 shows the DSC curves and the weight loss obtained for Co_2_Ni_2_Mg_2_Al_2_800 after DRM in the absence and presence of toluene. The exothermic peaks at 400–600 °C with weight losses in the same temperature range clearly demonstrate carbon oxidation. In fact, the weight loss that was recorded corresponds to the departure of easily oxidizable carbon species [33,50,51]. The amount of carbon is higher (higher weight loss) for Co_2_Ni_2_Mg_2_Al_2_800 T180 than for Co_2_Ni_2_Mg_2_Al_2_800. This result correlates with the TPO one. It is inferred that the presence of toluene increased the amount of carbon formed. Since the carbon balance is higher in the presence of toluene than in its absence (Figure 11), it is deduced that the higher amount of carbon on Co_2_Ni_2_Mg_2_Al_2_800 T180 is due to toluene cracking (reaction (g)). Furthermore, some authors who studied the effect of hydrocarbons on methane reforming attributed the decrease of the catalysts’ efficiency to the adsorption of contaminants on their active sites, thus modifying their catalytic properties and accelerating the carbon deposition [13,52]. Other authors have added to these causes the sintering of the metal particles [33,45] leading to a progressive catalyst deactivation. In our case, the lower amount of active metallic Ni and Co (XRD and TPO) and of MgO (XRD) detected for Co_2_Ni_2_Mg_2_Al_2_800 T180 could be ascribed to the adsorption of toluene or toluene-derived products on the catalyst sites. In fact, it is well known that the π bonds in the aromatics are in favor of their adsorption on solid materials. 

## 4. Conclusions

The mixed oxide Co_2_Ni_2_Mg_2_Al_2_800 material is more active and more stable (less deactivated by carbon) than a commercial catalyst Ni(50%)/Al_2_O_3_ in the dry reforming of methane despite the lower amount of Ni active sites in it. The interaction between Co and Ni, the better availability of Ni sites, and the higher specific surface area are responsible for these better catalytic performances. In addition, Co_2_Ni_2_Mg_2_Al_2_800 shows stability after 20 h under stream in dry reforming of methane. The good activity and stability are mainly attributed to a periodic cycle of carbon deposition and removal, and also to the strong interaction between Ni and Co.

In the presence of toluene in the reacting mixture, Co_2_Ni_2_Mg_2_Al_2_800 showed lower CH_4_ conversion and a higher amount of carbon. These behaviors are ascribed to hydrocarbon cracking reactions in addition to other side reactions that take place during reforming. Furthermore, competition between toluene and methane for the reaction with CO_2_ cannot be ruled out. The adsorption of hydrocarbons and the sintering of the particles could also be responsible for the deactivation of the catalyst in the presence of toluene. It is concluded that toluene decreases Co_2_Ni_2_Mg_2_Al_2_800 catalytic efficiency in terms of activity and stability. Therefore, more work has to be elaborated to enhance the catalyst properties toward the reforming of real biogas.
